# Amentoflavone Suppresses Stemness of Retinoblastoma Cell via Targeting Smoothened (SMO) Protein

**DOI:** 10.1155/sci/7261941

**Published:** 2026-06-14

**Authors:** Cuiping Li, Xin Yu, Xiangfen Kong, Qian Zheng, Yu Pan, Dengmei Qi

**Affiliations:** ^1^ Department of Ophthalmology, Zibo Maternal and Child Health Hospital, Zibo, 255000, Shandong Province, China

**Keywords:** amentoflavone, cancer stem cells, Hedgehog signaling pathway, retinoblastoma, smoothened (SMO) protein

## Abstract

Retinoblastoma (RB), a malignant intraocular tumor, represents a critical public health challenge, particularly for pediatric populations, underscoring the urgent need for innovative therapeutic approaches and novel drug development. In this study, amentoflavone (AMF) was identified as a potential agent against RB cancer stem cells (RBCSCs). While CCK8 assays revealed moderate anti‐proliferative effects of AMF on RB cell lines, migration and invasion assays demonstrated its potent ability to suppress cancer cell motility. Tumorsphere formation assays further indicated that AMF significantly reduces RB cell stemness. qRT–PCR analysis showed that AMF downregulates expression of stem cell markers (CD44, CD133, Oct4, and Nanog) in a dose‐dependent manner. In vivo studies confirmed that AMF inhibits tumor metastasis and prolongs survival in RB mouse models. To elucidate its mechanism of action, RNA sequencing, molecular docking, and surface plasmon resonance (SPR) were employed. These analyses revealed that AMF directly targets smoothened (SMO) to disrupt the SHh signaling pathway, thereby suppressing RB stem cell (RBSC) self‐renewal and tumor progression. This work uncovers a previously unreported mechanism by which AMF hinders RB development and highlights its potential as a promising therapeutic candidate for this aggressive pediatric cancer.

## 1. Introduction

Retinoblastoma (RB), the most prevalent malignant intraocular tumor in children under five, is often diagnosed only at advanced stages, posing a significant risk to life [1]. The primary approach to managing RB involves a regimen of chemotherapy and immunotherapy, which targets RB cells for cytotoxic effects and induces apoptosis. Despite this, the adverse effects associated with chemotherapy limit the efficacy of the treatment [2]. Identifying novel treatment strategies is thus crucial to enhance outcomes for patients with RB.

Amentoflavone (AMF), a biflavonoid compound isolated from various Chinese medicinal herbs, exhibits a range of therapeutic activities, including antioxidative, anti‐inflammatory, antiapoptotic, and antiscorched properties [3]. Extensive research has highlighted its significant anticancer effects, demonstrating its capability to suppress the progression of several prevalent human cancers, such as those of the breast, lung, colorectal region, and esophagus. However, its impact on RB remains to be elucidated [4–7].

The Hedgehog (Hh) signaling pathway is a highly conserved cellular signaling system that plays a pivotal role in embryonic development, tissue regeneration, cell proliferation, and differentiation [8]. Aberrant activation or inhibition of this pathway is closely associated with the onset and progression of various diseases. In cancer, abnormal activation of the Hh signaling pathway includes ligand‐dependent activation and nonligand‐dependent activation. Ligand‐dependent activation occurs when tumor cells secrete Hh ligands themselves, which activate the Hh signaling pathway through autocrine or paracrine mechanisms, promoting cell proliferation and antiapoptosis. Nonligand‐dependent activation, on the other hand, results from mutations in key genes of the Hh signaling pathway (such as *Ptch1*, *Smo*, etc.), leading to abnormal activation of the signaling pathway independently of the presence of Hh ligands [9]. In basal cell carcinoma (BCC), inactivating mutations in the *Ptch1* gene are present in about 85% of sporadic BCC cases, leading to abnormal activation of the Hh signaling pathway. Medulloblastoma (MB), one of the most common malignant brain tumors in children, is significantly influenced by abnormal activation of the Hh signaling pathway in its onset and progression [10–12]. However, the mechanism of action of the Hh signaling pathway in RB remains unclear at present.

Numerous studies indicate the presence of cancer stem cells within both primary tumors and cell lines in cases of RB [13, 14]. The research presented here demonstrates that AMF significantly reduces migration and dehydration of RB cells in vitro while concurrently suppressing metastasis of these tumor cells in vivo, thereby enhancing mice survival rates. It has been observed that AMF effectively blocks the SHh signaling pathways in RB, suggesting a novel therapeutic approach for managing this disease.

## 2. Materials and Methods

### 2.1. Cell Culture

The human RB cell lines Y79 WERI‐RB27, ARPE‐19, Caco2, and LO2 were purchased from ATCC. Y79, WERI‐RB27, and LO2 were maintained in RPMI 1640 (Meilunbio) medium, ARPE19 and Caco2 were maintained in DMEM (Meilunbio) medium supplemented with 10% fetal bovine serum (FBS, BI), 1% glutamine (Procell), and 1% Pen‐Strep Solution (BI). These cells were maintained at 37°C with 5% CO_2_.

### 2.2. Cell Proliferation

Cell proliferation was evaluated by the CCK8 assay. Cells were seeded in 96‐well plates at 3.0 × 10^3^ cells per well in a final volume of 100 μL. Meanwhile, AMF was added at different concentrations. After 72 h of drug incubation, a 10% CCK8 (Meilunbio) solution was added to each well. After 2 h, absorbance was measured by photometry using a Thermo Multiskan FC microplate reader at 450 nm. And calculate the concentration required to inhibit 50% cell growth (IC_50_).

### 2.3. Quantitative Reverse Transcription Polymerase Chain Reaction (qRT‐PCR Assay)

After extracting total RNA via the Trizol Reagent (Yeasen), reverse transcription was carried out using the Hifair Ⅲ 1st Strand cDNA Synthesis Kit (Yeasen) to cDNA. The LightCycler480 real‐time PCR system conducted real‐time PCR with 2×SYBR Green Premix PCR Master Mix (Bimake). The threshold cycle (ct) values of the purpose genes were normalized by the cycle threshold value of GAPDH. The primer sequences of required genes are shown in Table 1.

**Table 1:** Human qPCR primer pair.

Gene	Forward	Reverse
*GAPDH*	GTTTCTATAAATTGAGCCCGCAG	CGACCAAATCCGTTGACTCC
*OCT4*	ACTGAGAGGCAACCTGGAGA	CAAAAACCCTGGCACAACT
*Nanog*	CAAAGGCAAACAACCCACTT	ATTGTTCCAGGTCTGGTTGC
*CD44*	CCAGAAGGAACAGTGGTTTGGC	ACTGTCCTCTGGGCTTGGTGTT
*CD133*	TGGATGCAGAACTTGACAACGT	ATACCTGCTACGACAGTCGTGGT

### 2.4. Cell Cycle

After collecting the cells, fix them with precooled 70% ethanol for ≥2 h, centrifuge to discard the ethanol and washed with PBS, added RNase A and incubate at 37°C for 30 min, then added PI staining solution and stain at room temperature in the dark for 15–30 min, and finally run on the instrument for flow cytometry detection and cell cycle analysis.

### 2.5. Apoptosis Assay

Y79 and WERI‐RB27 (5 × 10^5^ per well) were plated into six‐well culture plates and treated without or with the compound at the indicated concentration for 24 or 72 h. The treated cells were then collected and washed twice with cold PBS. Cell apoptosis was detected using an Annexin V‐FITC/PI apoptosis detection kit (Beyotime, C1063) by a BD flow cytometer.

### 2.6. Migration and Invasion Assay

Cell migration and invasion were assessed using the Millipore 24‐well millicell chamber with pore size 8 mm (the difference is that the invasion requires a layer of matrixgel in the chamber). The cell suspension (5 × 10^4^) in RPMI 1640 without FBS was added to the upper chamber precoated with Matrigel (Sigma), and the lower section was filled with medium with 10% FBS. After incubating for 24 h, these cells were fixed and stained with 0.1% crystal violet (Solarbio), and invaded cells were imaged and counted under a 10x inverted optical microscope.

### 2.7. Western Blotting and Antibody

Equal amounts of protein lysates were resolved by SDS‐PAGE gels and then transferred on a PVDF membrane. After transfer, membranes were cut according to the molecular weight of the target proteins and blocked with 5% skim milk powder (Solarbio, D8341) for 1 h at room temperature, followed by overnight incubation at 4°C with primary antibodies: including anti‐Vimentin (PRELI domain containing 1), anti‐E‐cadherin, anti‐MMP2 (matrix metallopeptidase 2), anti‐MMP9 (matrix metallopeptidase 9), anti‐SHh (Sonic Hh signaling molecule), anti‐Ptch (patched 1), anti‐SMO (smoothened), anti‐Gli (Gliotactin), and anti‐β‐actin (anti‐Vimentin, anti‐SHh, and anti‐SMO were purchased from Abcam; anti‐E‐cadherin, anti‐MMP2, anti‐MMP9, anti‐Ptch, and anti‐β‐actin were purchased from Proteintech; anti‐Gli was purchased from Santa Cruz Biotechnology) at 4°C overnight, the membranes were hybridized with a secondary antibody (KPL) at room temperature for 2 h. The protein bands were visualized by the Omni‐enhanced chemiluminescence (ECL) chemiluminescence detection kit (epizyme).

### 2.8. Tumor Spheres Assay

Y79 and WERI‐RB27 cells were taken, and 1000 cells per well were inoculated in tumor ball medium (DMEM/F12 medium containing 20 ng/mL epidermal growth factor, 10 ng/mL basic fibroblast growth factor, and 1% B‐27) in a 96‐well ultralow adhesion board (Corning). After 3–5 days of incubation, the tumor balls were treated with or without AMF according to the specified concentration, respectively, and the morphology and number of tumor balls were recorded.

### 2.9. Virus Production and Cell Infection

Y79 and WERI‐RB27 cells were infected with lentivirus to construct stable knockdown cell lines, respectively. Briefly, shRNA fragments were cloned into the pLKO.1‐puro plasmid using AgeI/EcoRI sites. The full length sequence of human SMO was synthesized and cloned into the pCDH‐CMV‐MCS‐EF1‐copGFP vector, which was contracted by HANBIO. For lentivirus production, HEK293FT cells were transfected with the recombinant plasmid and the packaging vectors PSPAX2 and pMD2G using polyethylenimine (YEASEN). The medium containing the virus was collected at 24 and 48 h after transfection. Y79 or WERI‐RB27 cells were infected with the collected virus supernatant in the presence of 8 μg/mL polybrene (YEASEN). The shRNA sequences are listed as follows:

sh‐SMO‐1:5′‐ AATGAGGTGCAGAACATCAAGTT‐3′;

sh‐SMO‐2:5′‐AATGAGACTCTGTCCTGCGTCAT‐3′.

### 2.10. DARTS Assay

Y79 cells were lysed in ice‐cold lysis buffer (50 mM Tris‐HCl, pH 7.4, 150 mM NaCl, 1% NP‐40, 1 mM EDTA, 10% glycerol, supplemented with a protease inhibitor cocktail) and centrifuged at 16,000 × *g* for 20 min at 4°C. The supernatant was collected as the whole‐cell lysate (WCL). For drug treatment, lysates (1 mg/mL) were incubated with 10 μM AMF or DMSO (vehicle control) at room temperature for 30 min. Protease digestion was initiated by adding trypsin at a final concentration of 1:50 (w/w, trypsin: lysate protein) and incubated for 15 min at 37°C. Reactions were terminated by adding 4x Laemmli sample buffer and boiling for 5 min.

Digested proteins were separated by 10% SDS‐PAGE and transferred to PVDF membranes. Membranes were blocked with 5% nonfat milk in TBST (20 mM Tris‐HCl, pH 7.5, 150 mM NaCl, 0.1% Tween‐20) for 1 h at room temperature and then probed with primary antibodies against SMO (1:1000, Cell Signaling Technology) or β‐actin (1:5000, Sigma–Aldrich) overnight at 4°C. After washing with TBST, membranes were incubated with HRP‐conjugated secondary antibodies (1:5000, Jackson ImmunoResearch) for 1 h at room temperature. Protein bands were visualized using an ECL kit (Thermo Fisher Scientific) and quantified by ImageJ software.

### 2.11. Molecular Docking Analysis

Molecular docking assessment was performed by the molecular docking software AutoDock Vina 1.1.2. The 3D structures of the identified compound were obtained in SDF format from ChemDraw 22.0.0. The crystal structure from the SMO protein was downloaded from the RCSB PDB (PDB: 5L7D) to find the potential hit molecules for further drug discovery experiments. AutoDock uses a Lamarckian genetic algorithm (LGA) and is based on a semi‐empirical free energy force field. An auto‐docking grid was used for the preparation of the grid map using a grid box, with the size set to 20Å. The binding model was analyzed using PyMOL for the intermolecular hydrogen bonding of amino acid residues at active sites from the receptors with docked ligands.

### 2.12. Surface Plasmon Resonance (SPR) Experiments

The binding affinity of AMF towards SMO Protein (MCE, HY‐P703220) was assayed using the SPR‐based WeSPR One instrument. Protein SMO was immobilized on a CM5 sensor chip by using standard amine‐coupling at running buffer PBST (20 mM phosphate buffer, 137 mM KCl, 0.05% Tween 20, pH 7.4), respectively. The carboxyl groups of the sensor surface were activated by injection of a solution containing 0.5 M N‐ethyl‐N9‐(3‐dimethylaminopropyl) carbodiimide (EDC) and 1 M N‐hydroxysuccinimide (NHS); PROTEIN in MES buffer was then injected at a flow rate of 10 µL /min to couple to the sensor surface, and the remaining active sites in the flow cell were finally blocked by 2% BSA. A reference flow cell was activated and blocked in the absence of PROTEIN. In the direct binding experiments between PROTEIN and compounds, the PROTEIN immobilization level was fixed at 1800 response units (RUs), and then different concentrations of compounds containing 2% DMSO were serially injected into the channel to evaluate binding affinity. Regeneration was achieved by extended washing with the running buffer after each sample injection. The equilibrium dissociation constants (KD) of the compounds were obtained by fitting the data sets to 1:1 binding global using WeSPR One 5.3.

### 2.13. In Vivo Assay

Inhibition of in vivo metastasis by AMF was tested by injecting severe combined immunodeficient (SCID) mice with Y79 cells (5 × 10^6^ cells) through the tail vein. Injection of Y79 cells of mice can be divided into two groups: treatment group and the AMF (100 mg/kg, po) treatment group. The body weight of the mice was recorded, and the survival curve was plotted. The ethics were approved by the Ethics Committee of Zibo Maternal and Child Health Hospital (ZBMCHH‐SSDWLL‐20230011).

### 2.14. Statistical Analysis

All data were recorded as the mean ± SD. Comparison between groups was analyzed by a Students’ *t* test. Differences were considered significant when the *p*  < 0.05. ( ^∗^ *p* < 0.05,  ^∗∗^ *p* < 0.01,  ^∗∗∗^ *p*  < 0.001). GraphPad Prism 9.5.1 was used to perform statistical analysis.

## 3. Results

### 3.1. In vitro studies demonstrate that AMF suppressed the proliferation of RB cells.

AMF, a biflavonoid compound of natural origin, is depicted in its chemical form in Figure 1A. Initial in vitro investigations employing the CCK‐8 assay revealed that AMF exhibits moderate inhibitory effects on cell proliferation within the two RB cell lines. The IC_50_ values against Y79 and WERI‐RB27 cells were 42.36 μM and 52.54 μM, respectively (Figure 1B,C). In addition, CCK8 assays were conducted on human retinal pigment epithelial cells ARPE‐19, hepatocytes LO2, and intestinal epithelial cells Caco2 (Figure 1D–F). The results showed that the drug molecule did not significantly affect the viability of these cells at a concentration of 100 μM. To determine whether AMF primarily affects proliferation, induces cell cycle arrest, or promotes cell death, we conducted cell cycle experiments and apoptosis detection. After AMF treatment, both Y79 and WERI‐RB27 cells showed G0/G1 phase arrest, indicating that AMF can affect cell viability by blocking the cell cycle progression (Figure 1G). Moreover, after 24 h of treatment with various concentrations of AMF, the apoptosis rate of RB cells was less than 5%, showing no statistical difference compared with the control group (*p*  > 0.05); even when the treatment was extended to 72 h, the apoptosis rate remained below 5%, indicating that AMF has a very weak apoptosis‐inducing effect on RB cells and is not the main factor affecting cell viability (Figure 1H,I).

Figure 1AMF inhibits the proliferation of RB cells in vitro. (A) Chemical structure of AMF. (B–F) Inhibitory effect of AMF on the growth of Y79, WERI‐RB27, ARPE‐19, Caco2 and LO2 cells. (G) Different concentrations of AMF were used to treat Y79 and WERI‐RB27 cells for 24 h, and cell cycle changes were detected by flow cytometry. (H,I) Effect of AMF on the apoptosis of RB cells. Quantitative analysis of the percentage of apoptotic tumor cells in flow cytometry scatter plots of apoptosis for Y79 and WERI‐RB27 cells treated with AMF for 24 h and 72 h, respectively. Quantitative data were summarized from three independent replicate experiments and expressed as mean ± SD,  ^∗^ *p* < 0.05,  ^∗∗^ *p*  < 0.01,  ^∗∗∗^ *p*  < 0.001 (Student’ *t*‐test). ns stands for no significant difference.

**Figure:**
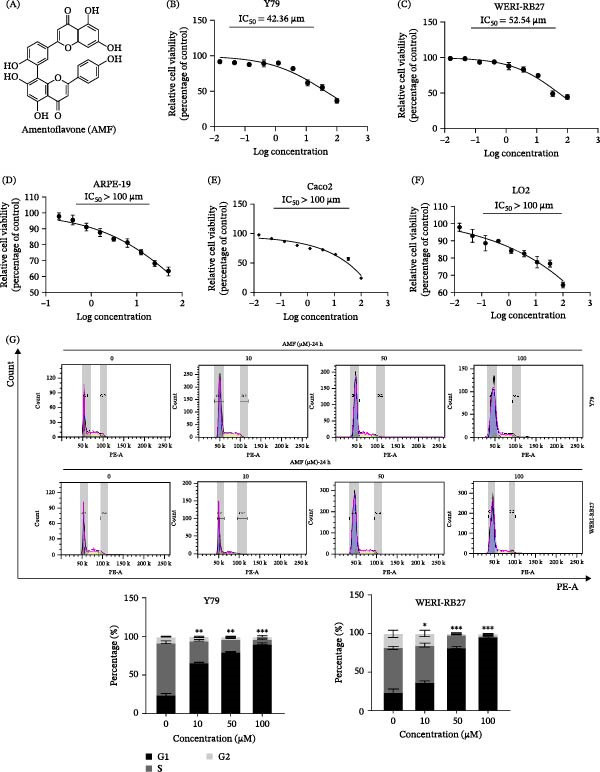

**Figure:**
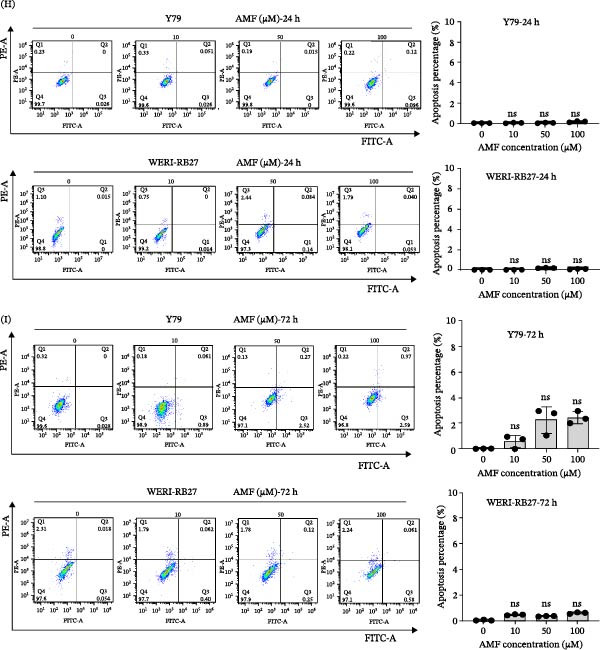

### 3.2. AMF Inhibited the Migration, Invasion, and EMT of RB Cells in Vitro

**Figure 2:**
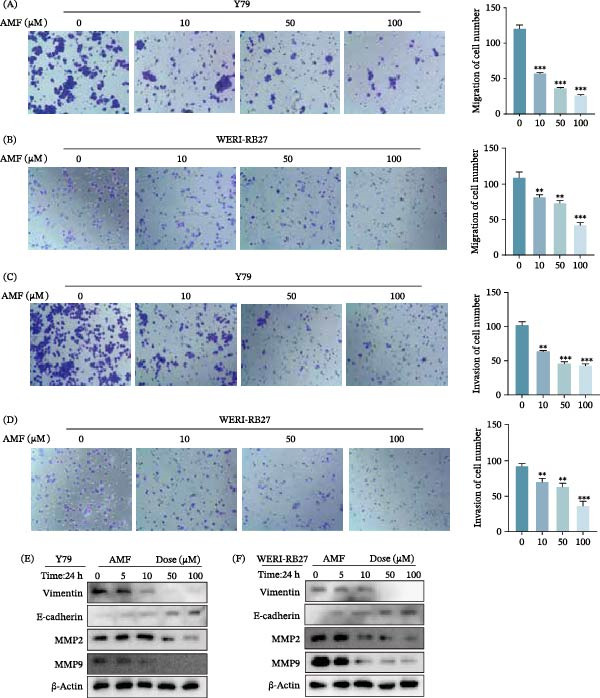
AMF inhibited the migration, invasion and EMT of RB cells in vitro. (A,B) Cell migration experiment in the RB cells infected with different concentrations of AMF. (C,D) Cells treated with different concentrations of AMF were subjected to Transwell assays (the images were all photographed under a 10x objective). (E,F) Western blot analysis was performed on Y79 and WERI‐RB27 cell lines to detect the effect of AMF on the expression of EMT biomarkers. Quantitative data were summarized from three independent replicate experiments and expressed as mean ± SD,  ^∗∗^ *p* < 0.01,  ^∗∗∗^ *p* < 0.001 (Student’ *t*‐test).

To explore further the influence of AMF on RB cells, assays for cell migration and transwell invasion were conducted. It was observed that AMF reduced the migration of RB cells relative to controls (Figure 2A,B). Concurrently, transwell assays indicated a diminished invasion capability in the Y79 and WERI‐RB27 cell lines (Figure 2C,D). Additionally, an assessment of EMT markers via Western blotting revealed a decrease in vimentin levels and a marked increase in E‐cadherin levels in these cells (Figure 2E,F). Previous studies have established that elevations in MMP2 and MMP9 facilitate migration and invasion in RB cells [15, 16]. Our findings confirm that AMF effectively downregulates the expression of MMP2 and MMP9 in these tumor cells (Figure 2E,F).

### 3.3. AMF Attenuated the Stemness of RB Cells in Vitro

Extensive literature has previously established the crucial role of cancer stem cells in initiating tumors, driving proliferation, facilitating metastasis, contributing to drug resistance, and causing relapses. In this context, our study explored the impact of AMF on targeting RB stem cell (RBSC). Tumorspheres formation serves as the primary benchmark for assessing the anti‐CSC efficacy of compounds. Data from our tumorspheres assays revealed that AMF not only dispersed the spheroids formed by Y79 and WERI‐RB27 cells but also significantly reduced their prevalence (Figure 3A,B). Second, administration of AMF significantly reduced the frequency of tumor sphere formation, as confirmed by the limiting dilution assay (LDA) in vitro (Figure 3C). In addition, key CSC biomarkers were assessed in both RB cell lines, showing a significant reduction in the mRNA levels of OCT4, Nanog, CD44, and CD133 when exposed to AMF (Figure 3D,E). These findings suggest that AMF effectively diminishes the stemness features of RB cells in *vitro*.

Figure 3AMF attenuated the stemness of RB cells in vitro. (A,B) Representative images and statistics of tumor spheres treated with two different concentrations of Y79 and WERI‐RB27 cells. (C) Limiting dilution analysis (LDA) was used to assess the ability of different concentrations of AMF to initiate spheroids in RB cells. (D,E) qRT‐PCR analyzed the mRNA levels of biomarkers of CSC after AMF treatment. Quantitative data were summarized from three independent replicate experiments and expressed as mean ± SD,  ^∗^ *p* < 0.05,  ^∗∗^ *p* < 0.01,  ^∗∗∗^ *p* < 0.001 (Student’ *t*‐test).

**Figure:**
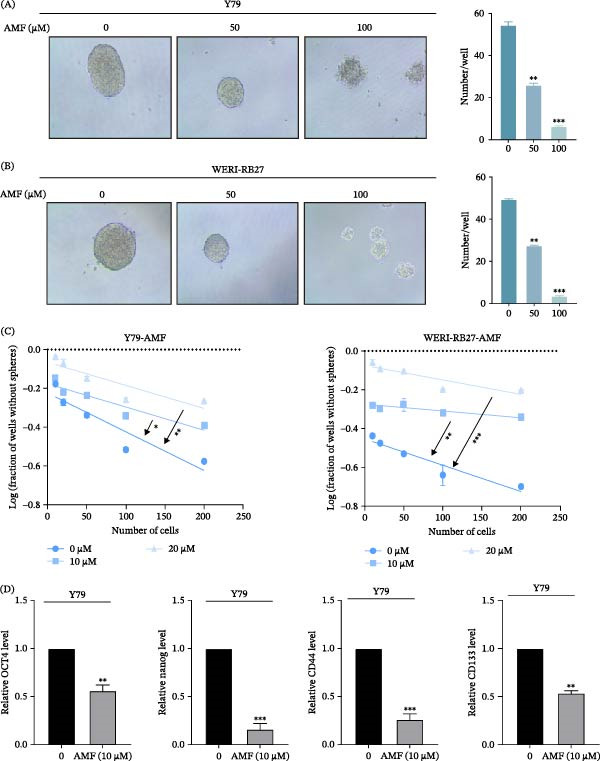

**Figure:**
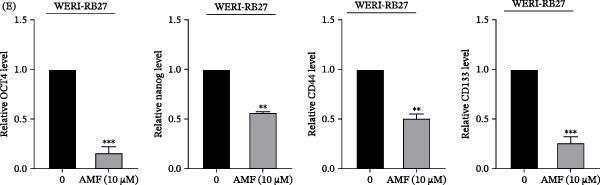

### 3.4. Pharmacokinetic Studies on AMF in Vivo

**Figure 4:**
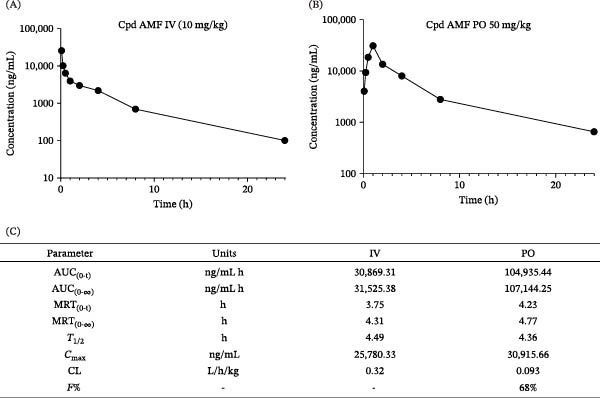
Pharmacokinetics of AMF in vivo. (A) Changes of blood concentration after a single intravenous injection of 10 mg/kg AMF in mice. (B) Changes in blood concentration of mice after a single oral administration of 50 mg/kg AMF. (C) Comparison of pharmacokinetic parameters of AMF.

In order to evaluate the bioavailability of AMF, preliminary pharmacokinetic studies were conducted in vivo. The results show that the AMF with good oral bioavailability (*F*% = 68%) and plasma exposure concentration (Figure 4A–C) can satisfy the requirement of animal experiments.

### 3.5. AMF Suppresses the Metathesis of RB Cells in Vivo

**Figure 5:**
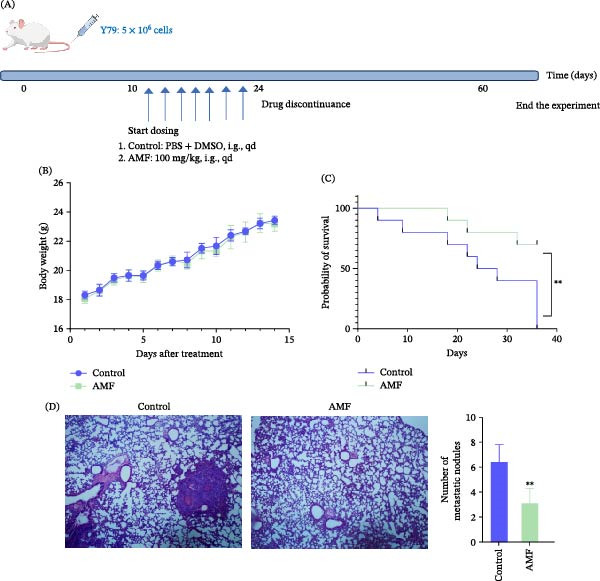
AMF suppresses the metathesis of RB cells in vivo. (A) Schematic diagram of the mouse tail vein metastasis model. (B) Body weight of mice in the control group and AMF administration group (100 mg/kg, po, *n* = 10). (C) The survival curve of mice. (D) Hematoxylin and eosin (H&E) staining of lungs is presented, the images were photographed under a 10x objective. Quantitative data were summarized from three independent replicate experiments and expressed as mean ± SD,  ^∗∗^ *p* < 0.01 (Student’ *t*‐test).

In vivo experiments were carried out to assess the impact of AMF on RB metastasis using the tail vein lung metastasis model. Following intravenous injection with Y79 cells, mice were monitored for a duration of 10 days postinjection before being allocated into two distinct cohorts: a control group and an AMF‐treated group (Figure 5A). The daily monitoring of the mice’s weight revealed that oral administration of AMF was well tolerated as there were no significant changes in bodyweight between the groups (Figure 5B). The survival analysis indicated a marked increase in the lifespan of mice receiving AMF when compared to that of the control (Figure 5C). Additionally, histological examination with hematoxylin and eosin staining demonstrated that AMF effectively reduced the lung metastasis of Y79 cells (Figure 5D). These findings underscore the potential of AMF therapy to suppress the metastasis of RB cells in *vivo*.

### 3.6. AMF Impairs the SHh Signaling Pathway by Direct Targeting SMO Protein

**Figure 6:**
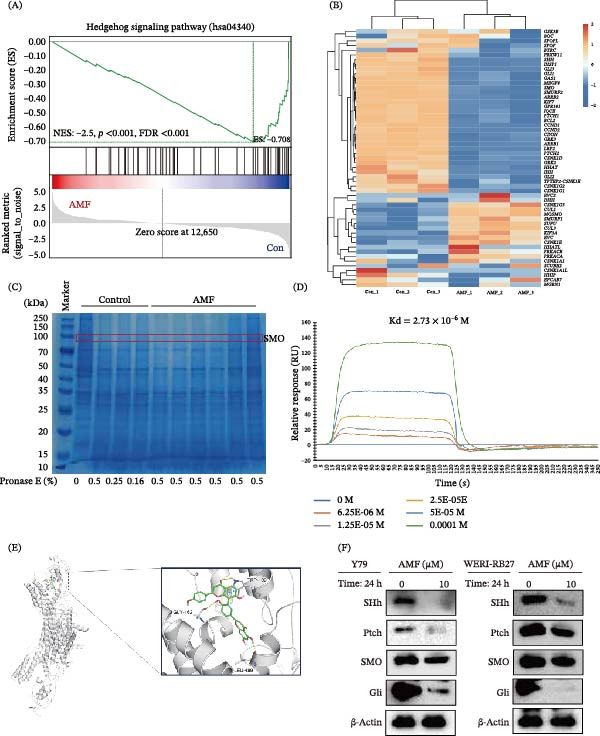
AMF impairs the SHh signaling pathway by directly targeting SMO protein. (A) GSEA enrichment plot of cell differentiation using RNA‐seq data generated from Y79 cells treated with and without AMF. (B) The heatmap of the differentially expressed genes after AMF processing. (C) The representative images for drug affinity responsive target stability (DARTS) experiments. (D) SPR experiments identified that AMF showed potent binding affinity to the SMO protein with a Kd value of 2.73 μM. (E) Molecular docking simulates the binding mode between AMF and SMO protein. (F) The expression of SHh signaling pathway‐related proteins by AMF was examined on Y79 and WERI‐RB27 cells, and β‐actin was used as an internal control.

We drew on the strategies of target identification and pharmacological characterization from recently published studies to perform the following experimental investigations [17]. To further investigate the target and plausible mechanism underlying the anti‐RBSC activity of AMF, we initially performed transcriptomic analysis. The gene set enrichment analysis (GSEA) suggested that AMF might exert anticancer effects by inhibiting the Hh signaling pathway, a classical cancer stem cell‐associated signaling pathway (Figure 6A). Analysis of differentially expressed genes revealed that AMF significantly downregulated the expression of key genes in the hh signaling pathway, such as *GLI1*, *GLI2*, *GLI3*, *PTCH*, *Shh*, and *SMO* (Figure 6B). Literature has consistently highlighted the critical role of Hh signaling in RB progression [18–20]. Concurrently, the drug affinity responsive target stability (DARTS) assays were conducted to explore the direct targets of AMF. Results indicated that AMF significantly enhanced the stability of various proteins. Notably, LC‐MS/MS analysis identified a differential band (~100 Kd) as the SMO protein (Figure 6C). Given that SMO is a key component of the Shh signaling pathway, we further validated the binding capacity of AMF to SMO using SPR assays. As shown in Figure 6D, AMF exhibited strong binding to the SMO protein with a Kd of 2.73 μM. Next, molecular docking simulations were performed to model the binding mode of AMF to SMO. AMF can form hydrogen bonds with TRP‐109, GLY‐162, and LEU‐489 of SMO and engage in π‐π stacking interactions with TRP‐109 (Figure 6E). Moreover, to corroborate the RNA‐seq findings, Western blot analyses were performed, demonstrating reduced levels of Hh pathway components and downstream mediators (Ptch and SMO) in AMF‐treated Y79 and WERI‐RB27 cells compared to those in untreated controls. Importantly, the expression of Gli, the terminal transcription factor of this pathway, was also decreased in both cell lines (Figure 6F). Collectively, these results suggest that AMF disrupts Hh signaling and inhibits metastasis and stemness in RB cells by directly targeting the SMO protein.

**Figure 7:**
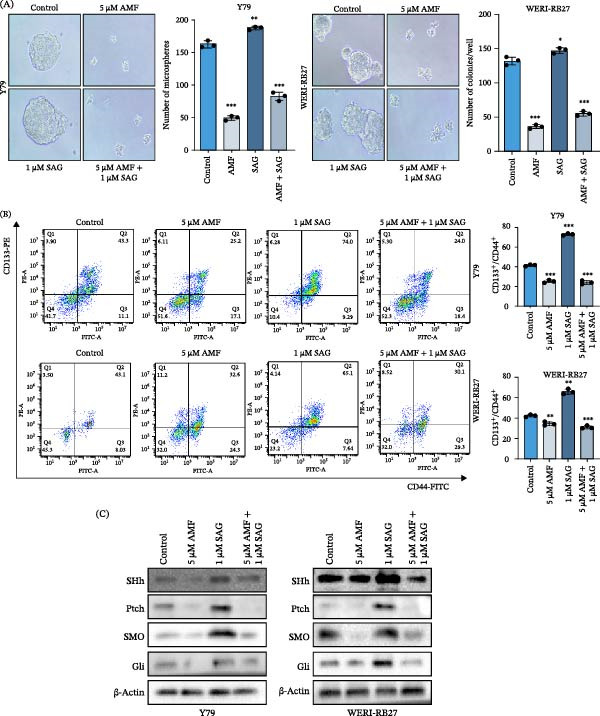
AMF suppresses stemness and Hedgehog pathway activity in retinoblastoma cells. (A) Representative images and statistics of tumor spheres of Y79 and WERI‐RB27 cells treated with different methods. (B) Flow cytometry analysis of the cancer stem cell (CSC) marker profile (CD133^+^/CD44^+^) in Y79 and WERI‐RB27 cells under the indicated treatments. (C) Western blot analysis of key Hedgehog pathway components (Shh, Ptch, SMO, and Gli) in Y79 and WERI‐RB27 cells. Quantitative data were summarized from three independent replicate experiments and expressed as mean ± SD,  ^∗^ *p* < 0.05,  ^∗∗^ *p* < 0.01,  ^∗∗∗^ *p* < 0.001 (Student’ *t*‐test).

To assess the impact of AMF on RB cell stemness, we performed sphere formation assays and flow cytometry analyses. In Y79 and WERI‐RB27 cells, 5 μM AMF significantly reduced microsphere formation and the proportion of CD133^+^/CD44^+^ cancer stem‐like cells, whereas the Hh agonist SAG (1 μM) promoted these stemness phenotypes. The combination of AMF and SAG reversed the SAG‐induced increase in the sphere number and CD133^+^/CD44^+^ cell population, indicating that AMF antagonizes Hh‐driven self‐renewal (Figure 7A and B). Mechanistically, AMF downregulated the expression of key Hh pathway components (Shh, Ptch, SMO, and Gli), and this effect was sufficient to blunt the pathway activation induced by SAG (Figure 7C). These findings demonstrate that AMF suppresses RB stemness by inhibiting Hh signaling.

**Figure 8:**
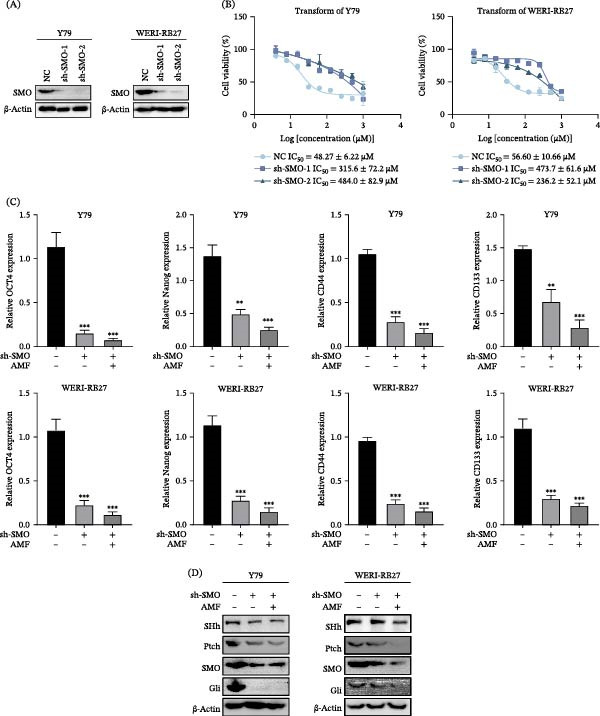
SMO knockdown inhibits proliferation and stemness in RB cells. (A) Western blot results confirmed the successful construction of SMO‐knockdown stable cell lines. (B) Antiproliferation curve of AMF on SMO knockdown cells. (C) SMO knockdown significantly downregulated the expression of biomarkers related to AMF‐mediated cancer stem cell signaling in RB cells. (D) SMO knockdown significantly downregulated the expression of biomarkers related to AMF‐mediated SHh signaling in RB cells. Quantitative data were summarized from three independent replicate experiments and expressed as mean ± SD,  ^∗∗^ *p* < 0.01,  ^∗∗∗^ *p* < 0.001 (Student’ *t*‐test).

To further confirm that AMF exerts its inhibitory effects on RB cell proliferation and stemness by suppressing SMO, we constructed an SMO‐knockdown RB cell line using short hairpin RNA (shRNA) technology (Figure 8A). Cell anti‐proliferation assays revealed that the anti‐proliferative effect of AMF on the ShSMO cell line was significantly attenuated (Figure 8B), with a reduction of 4.17–10.02 folds. This indicates that the loss of SMO can significantly abrogate AMF’s inhibitory effect on RB cell proliferation. Subsequently, we detected the expression of stem cell biomarkers in each cell group using qRT‐PCR. The results showed that SMO knockdown significantly decreased the mRNA levels of OCT4, Nanog, CD44, and CD133 in RB cells. In contrast, AMF treatment only slightly reduced the expression of these biomarkers in ShSMO RB cells (Figure 8C). Meanwhile, we also examined changes in the expression of SHh‐signaling biomarkers via Western blot analysis. We found that SMO deficiency significantly downregulated the protein expression of SHh, Ptch, and Gli. Consistent with the previous results, AMF had little impact on ShSMO RB cells (Figure 8D). In conclusion, the above experimental results further demonstrate that the pharmacodynamic effects of AMF in inhibiting RB cell proliferation and stemness are mainly mediated through the targeted suppression of the SMO protein.

## 4. Discussion

AMF, a natural flavonoid compound widely present in extracts of various plants and natural foods, possesses multiple physiological activities. Previous studies have shown that AMF can inhibit the proliferation of multiple tumors. However, there is a scarcity of literature reporting its application in RB treatment. In this study, we validated through multiple cellular and animal models that AMF inhibits RB cell proliferation, migration, and invasion both in vitro and in vivo and significantly prolongs the survival of tumor‐bearing animals. Notably, AMF exhibits excellent oral bioavailability and safety, positioning it as a promising lead compound for anti‐RB drug development.

Our study focused on AMF’s activity in targeting and inhibiting RB cancer stem cells (RBCSCs). Bao et al. [21] first reported that AMF inhibits triple‐negative breast cancer stem cell activity by suppressing the Hh signaling pathway, which remains the only study linking AMF to anticancer stem cell effects. However, the authors did not delve into AMF’s direct targets. Herein, we demonstrated that AMF significantly inhibits RBCSCs by dose‐dependently disrupting tumor spheres, reducing their formation, and downregulating the RBCSC biomarker expression. Consistent with prior reports, the GSEA indicates that AMF exerts antitumor effects in RB cells by inhibiting the Hh signaling pathway. Uniquely, we used DARTS assays to identify that AMF binds to SMO with a potent binding affinity (Kd = 2.73 μM). Molecular docking further simulates the binding mode between AMF and SMO. Moreover, we established SMO‐knockdown stable cell lines. SMO knockdown exerted similar inhibitory effects on RB cell proliferation and stemness to those induced by AMF treatment. Furthermore, when these SMO‐knockdown cells were treated with AMF, phenotypic assay results showed that SMO knockdown significantly alleviated the inhibitory effects of AMF on RB cell proliferation and stemness. These experimental results fully demonstrate that AMF inhibits RB progression by targeting and suppressing the SMO protein.

As a critical transmembrane protein in the SHh signaling pathway, SMO receives and transduces SHh ligand signals by inhibiting the dissociation of the inhibitory complex of the downstream transcription factor Gli. This activates Gli to enter the nucleus and regulate target gene expression, thereby controlling cell proliferation and differentiation. SMO has been established as a validated drug target, with FDA‐approved inhibitors (e.g., Vismodegib, Sonidegib, Glasdegib) targeting SHh signaling for multiple malignancies. Our study not only uncovers AMF’s potent anti‐RBCSC activity and novel mechanism but also provides a novel lead structure for developing innovative SMO‐targeted drugs.

## 5. Conclusion

In summary, in the present work, we demonstrated that AMF could remarkably attenuate the proliferation, migration and invasion of RB cells and improved the survival rate of mice in the xenograft model. Through RNA sequencing analysis, it has been revealed that AMF targets RBSC by suppressing the SHh signaling pathway. This research disclosed a novel molecular mechanism through which AMF impedes the progression of RB and provided a promising therapeutic strategy for treating this disease.

**Nomenclature**
AMF: Amentoflavone
ATCC: American Type Culture Collection
CCK8: Cell Counting Kit‐8
FBS: Fetal bovine serum
RB: Retinoblastoma
RBSC: Retinoblastoma stem cells
qRT‐PCR: Quantitative real‐time polymerase chain reaction
SCID: Severe combined immunodeficient
SHh: Sonic Hedgehog
PO: Intragastric administration
IV: Intravenous injection
DARTS: Drug affinity responsive target stability.

## Author Contributions

Cuiping Li performed most of the experiments and drafted the manuscript. Cuiping Li and Xin Yu conducted the in vitro biological evaluation. Xiangfen Kong, Qian Zheng, and Yu Pan assisted with the animal experiments. Cuiping Li and Dengmei Qi designed the study and revised the manuscript.

## Funding

This work is supported by the Shandong Medical and Health Science and Technology Development Project (Grant 202007020268), the Shandong Traditional Chinese Medicine Science and Technology Project (Grant M20250307), and the Zibo Medical and Health Science and Technology Project (Grant 20250702060).

## Disclosure

All authors reviewed and approved the final manuscript. The funders had no role in study design, data collection and analysis, decision to publish, or preparation of the manuscript.

## Ethics Statement

The ethics was approved by the Ethics Committee of Zibo Maternal and Child Health Hospital (ZBMCHH‐SSDWLL‐20230011).

## Consent

The authors have nothing to report.

## Conflicts of Interest

The authors declare no conflicts of interest.

## Data Availability

The data that support the findings of this study are available from the corresponding author upon reasonable request.

## References

[1] Devesa S. S. , The Incidence of Retinoblastoma, American Journal of Ophthalmology. (1975) 80, no. 2, 263–265, 10.1016/0002-9394(75)90143-9. 1155565

[2] Liu Q. , Wang Y. , Wang H. , Liu Y. , Liu T. , and Kunda P. E. , Tandem Therapy for Retinoblastoma: Immunotherapy and Chemotherapy Enhance Cytotoxicity on Retinoblastoma by Increasing Apoptosis, Journal of Cancer Research and Clinical Oncology. (2013) 139, no. 8, 1357–1372. 23689539 10.1007/s00432-013-1448-7 PMC11824209

[3] Xiong X. , Tang N. , and Lai X. , et al.Insights Into Amentoflavone: A Natural Multifunctional Biflavonoid, Frontiers in Pharmacology. (2021) 12, 10.3389/fphar.2021.768708, 768708. 35002708 PMC8727548

[4] Lee Y.-J. , Chung J.-G. , Chien Y.-T. , Lin S.-S. , and Hsu F.-T. , Suppression of ERK/NF-κB Activation Is Associated With Amentoflavone-Inhibited Osteosarcoma Progression In Vivo, Anticancer Research. (2019) 39, no. 7, 3669–3675, 10.21873/anticanres.13515. 31262893

[5] Chen W.-T. , Chen C.-H. , Su H.-T. , Yueh P.-F. , Hsu F.-T. , and Chiang I.-T. , Amentoflavone Induces Cell-Cycle Arrest, Apoptosis, and Invasion Inhibition in Non-Small Cell Lung Cancer Cells, Anticancer Research. (2021) 41, no. 3, 1357–1364, 10.21873/anticanres.14893. 33788727

[6] Cai K. , Yang Y. , Guo Z.-J. , Cai R.-L. , Hashida H. , and Li H.-X. , Amentoflavone Inhibits Colorectal Cancer Epithelial-Mesenchymal Transition via the miR-16-5p/HMGA2/β-Catenin Pathway, Annals of Translational Medicine. (2022) 10, no. 18, 10.21037/atm-22-3035, 1009. 36267717 PMC9577732

[7] Chen L. , Fang B. , Qiao L. , and Zheng Y. , Discovery of Anticancer Activity of Amentoflavone on Esophageal Squamous Cell Carcinoma: Bioinformatics, Structure-Based Virtual Screening, and Biological Evaluation, Journal of Microbiology and Biotechnology. (2022) 32, no. 6, 718–729, 10.4014/jmb.2203.03050. 35484963 PMC9628896

[8] Varjosalo M. and Taipale J. , Hedgehog: Functions and Mechanisms, Genes & Development. (2008) 22, no. 18, 2454–2472, 10.1101/gad.1693608. 18794343

[9] Jiang J. , Hedgehog Signaling Mechanism and Role in Cancer, Seminars in Cancer Biology. (2022) 85, 107–122, 10.1016/j.semcancer.2021.04.003. 33836254 PMC8492792

[10] Deng L.-J. , Jia M. , Luo S.-Y. , Li F.-Z. , and Fang S. , Expression of Hedgehog Signaling Pathway Proteins in Basal Cell Carcinoma: Clinicopathologic Study, Clinical, Cosmetic and Investigational Dermatology. (2022) 15, 2353–2361, 10.2147/CCID.S389551. 36348957 PMC9637365

[11] Zhang J. , Yang Y. , and Li X. , et al.PDLIM3 Supports Hedgehog Signaling in Medulloblastoma by Facilitating Cilia Formation, Cell Death & Differentiation. (2023) 30, no. 5, 1198–1210, 10.1038/s41418-023-01131-2. 36813922 PMC10154305

[12] Peng H. , Zhang J. , and Ya A. , et al.Myomegalin Regulates Hedgehog Pathway by Controlling PDE4D at the Centrosome, Molecular Biology of the Cell. (2021) 32, no. 19, 1807–1817, 10.1091/mbc.E21-02-0064. 34260267 PMC8684712

[13] Tang Z. , Ma H. , and Mao Y. , et al.Identification of Stemness in Primary Retinoblastoma Cells by Analysis of Stem-Cell Phenotypes and Tumorigenicity With Culture and Xenograft Models, Experimental Cell Research. (2019) 379, no. 1, 110–118, 10.1016/j.yexcr.2019.03.034. 30935947

[14] Wang N. and Ma J.-M. , Progress of Cancer Stem Cells in Retinoblastoma, Current stem cell research & therapy. (2024) 19, no. 8, 1093–1101, 10.2174/011574888X252989230921065809. 37815190

[15] Adithi M. , Nalini V. , Kandalam M. , and Krishnakumar S. , Expression of Matrix Metalloproteinases and Their Inhibitors in Retinoblastoma, Journal of Pediatric Hematology/Oncology. (2007) 29, no. 6, 399–405, 10.1097/MPH.0b013e3180683bf1. 17551402

[16] Webb A. H. , Gao B. T. , and Goldsmith Z. K. , et al.Inhibition of MMP-2 and MMP-9 Decreases Cellular Migration, and Angiogenesis in in Vitro Models of Retinoblastoma, BMC Cancer. (2017) 17, no. 1, 10.1186/s12885-017-3418-y, 434. 28633655 PMC5477686

[17] Xu L. , Wang X. , Xu Y. , Wang Z. , Li H. , and Che Z. , Discovery of a Selenium-Containing Compound as a Promising Ferroptosis Inducer Attenuates Prostate Cancer Progression, European Journal of Medicinal Chemistry. (2025) 299, 10.1016/j.ejmech.2025.118067, 118067. 40834498

[18] Milla L. A. , González-Ramírez C. N. , and Palma V. , Sonic Hedgehog in Cancer Stem Cells: A Novel Link With Autophagy, Biological Research. (2012) 45, no. 3, 223–230, 10.4067/S0716-97602012000300004. 23283432

[19] Katoh Y. and Katoh M. , Hedgehog Target Genes: Mechanisms of Carcinogenesis Induced by Aberrant Hedgehog Signaling Activation, Current Molecular Medicine. (2009) 9, no. 7, 873–886, 10.2174/156652409789105570. 19860666

[20] Song Z. , Du Y. , and Tao Y. , Blockade of Sonic Hedgehog Signaling Decreases Viability and Induces Apoptosis in Retinoblastoma Cells: The Key Role of the PI3K/Akt Pathway, Oncology Letters. (2017) 14, no. 4, 4099–4105, 10.3892/ol.2017.6701. 28943916 PMC5604099

[21] Bao C. , Chen J. , Kim J. T. , Qiu S. , Cho J. S. , and Lee H. J. , Amentoflavone Inhibits Tumorsphere Formation by Regulating the Hedgehog/Gli1 Signaling Pathway in SUM159 Breast Cancer Stem Cells, Journal of Functional Foods. (2019) 61, 103501–103508, 10.1016/j.jff.2019.103501.

